# Case of Strangulated Femoral Hernia in the Male, Requiring Operation

**Published:** 1827-11

**Authors:** 

**Affiliations:** St. Thomas's Hospital.


*Case of Strangulated Femoral Hernia in the Male, requiring Operation, successfully treated by* Mr. Green, at St. Thomas's Hospital.

July 12th, 1827.—Thomas Steers, ætat. thirty-seven, a labouring man, was admitted with a femoral hernia on the right side. Some weeks prior to admission, he had laboured under typhous fever, from which he was now only convalescent; and on the 11th, during a sudden attack of sickness, he stated that he felt something give way in the right groin, and where, on examination, he found a swelling, which had not previously particularly drawn his attention, although, on recollection, he said he had noticed a swelling in that situation some time since.

The tumor is about the size of a pullet's egg, elastic and tender, as is also the abdomen in a slight degree. There is little anxiety expressed by the countenance; pulse moderately full and compressible; tongue white and coated; the bowels have not been open since the morning of the 11th. For the last few hours the stomach has been tranquil. Prior to admission, some aperient medicine was administered, but immediately rejected. He was then bled, but not to syncope, and the taxis employed for some time.

After his arrival in the hospital, he was placed in the warm bath, and the taxis again resorted to, without success. Mr. Green was then sent for, and shortly afterwards, finding other means ineffectual, proceeded to the operation. In dividing the integuments, a branch of the external pudic artery was cut, the extremities of which required ligatures; after laying open the sac, and exposing a small portion of dark-coloured small intestine, the stricture was divided, and the bowel easily replaced, when a copious discharge of serum followed. Immediate relief was experienced, and shortly after his return to bed the pulse acquired more fulness.

July 13th.—Has slept a little during the night; countenance natural; pulse eighty-four, with some tone; tongue moist; bowels have been twice open, but the fæces are small in quantity; abdomen but slightly painful on pressure.

℞. Magnes. Sulph. ʒij. ex Mist. Menth. secundâ quâque horâ donec alvus dejicerit.

After the second dose, the bowels were freely relieved, and the patient proceeded favourably in every respect. The ligatures came away on the eleventh day; and by the 6th of August he was restored to tolerably good health, and, having been provided with a truss, he shortly afterwards left the hospital.

---

